# Identification and evaluation of novel methods for the analysis of ordinal data in acute stroke trials in comparison to ordinal logistic regression

**DOI:** 10.1186/1745-6215-16-S2-P131

**Published:** 2015-11-16

**Authors:** Aimie Nunn, Laura Gray

**Affiliations:** 1 MRC Clinical Trials Unit, University College London, London, UK; 2 Department of Health Sciences, University of Leicester, Leicester, UK; 3 Leicester Clinical Trials Unit, University of Leicester, Leicester, UK

## Background

Ordinal outcomes are common; however, selecting an appropriate analysis is often problematic. Acute stroke trials routinely measure dependency using an ordinal scale as the primary outcome. Historically trials have dichotomised ordinal scales to compare the proportion dependent across groups, which limits statistical power to detect an effect. The OAST Collaboration (2007) showed ordinal logistic regression increased statistical power. Alternative methodologies for ordinal data proposed since 2007 were compared to ordinal logistic regression (OLR).

## Methods

PubMed and conference proceedings were searched for novel methods for analysis of ordinal outcome scales in stroke. Identified statistical methods were applied to data from the International Stroke Trial which randomised 19,435 patients to receive no treatment, aspirin, heparin or aspirin and heparin. All identified methods were compared to OLR in detection of the effect of aspirin versus no aspirin.

## Results

Two regression based techniques were described for the analysis of ordinal scales: partial proportional odds and adjacent categories models. Two methods were proposed using prognosis based cut points on ordinal scales: sliding dichotomy and trichotomy. Three absolute measures of treatment effect were identified: permutation test, number needed to treat (NNT) for ordinal data and win ratio. None of the methods assessed were observed to increase the power to find a treatment effect compared to OLR.

## Conclusions

None of the recently proposed methods appear to offer statistical advantage over OLR. However, reporting an absolute measure of effect such as the NNT alongside the results from OLR aids interpretability of the common odds ratio.

